# Azithromycin mitigates human rhinovirus impact on barrier integrity and function in non-diseased airway epithelium

**DOI:** 10.3389/fcell.2025.1532656

**Published:** 2025-06-26

**Authors:** Kevin Looi, Erika N. Sutanto, Thomas Iosifidis, Luke J. Berry, Anthony Kicic, Stephen M. Stick

**Affiliations:** ^1^ Wal-yan Respiratory Research Centre, The Kids Research Institute Australia, The University of Western Australia, Nedlands, WA, Australia; ^2^ School of Population Health, Curtin University, Bentley, WA, Australia; ^3^ Centre for Child Health Research, The University of Western Australia, Nedlands, WA, Australia; ^4^ Centre for Cell Therapy and Regenerative Medicine, School of Medicine and Pharmacology, The University of Western Australia and Harry Perkins of Institute of Medical Research, Nedlands, WA, Australia; ^5^ Department of Respiratory and Sleep Medicine, Perth Children’s Hospital, Nedlands, WA, Australia

**Keywords:** airway epithelium, tight junction, human rhinovirus, azithromycin, barrier function

## Abstract

**Introduction:**

Azithromycin improves symptomology in various chronic airway diseases exacerbated by viral infections. However, the mechanisms underlying the apparent antiviral effects of azithromycin remain unclear.

**Methods:**

Airway epithelial cells from healthy children were cultured, expanded and differentiated into air-liquid interface cultures. Submerged and differentiated primary cultures were treated with 10 µM of AZM for 24 h and subsequently infected with human rhinovirus (HRV)-1b for 24 h. Virus receptor expression, replication, progeny release and inflammatory cytokines (IL-1β, −6, −8 and IP-10) were then measured. Barrier integrity was determined via qPCR, in-cell western (ICW), immunofluorescence confocal microscopy, confocal microscopy, transepithelial electrical resistance (R_T_) measurement and an apparent permeability (P_*app*_) assay.

**Results:**

Treatment with AZM for 24 h at the concentrations of 0.1, 1 and 10 µM did not have any significant impact on either cellular viability or cytotoxicity in un-infected cells. No significant effect on viral receptor, cytokine expression was observed in non-infected cells treated with 10 µM AZM. Similarly, there was no significant change in both occludin and ZO-1 expression in non-infected cells. However, *claudin-1* gene expression was significantly reduced but corresponding protein expression was significantly increased following 10 µM AZM. Although R_T_ was significantly lower, this was not corroborated by any significant change in epithelial permeability after 10 µM AZM treatment. Subsequent to HRV-1b infection, 10 µM AZM treatment significantly reduced cytotoxicity induced by infection. Viral receptor expression were not affected with AZM pre-treatment but a significant decrease in viral replication was observed. Except for IP-10, expression of IL-1β, −6, and −8 was significantly reduced. Gene and protein expression of key epithelial junctions were significantly higher in treated, infected cells, which were concomitant with epithelial barrier function.

**Discussions:**

This study identified that AZM can protect against HRV-1b-induced epithelial damage. Our data, demonstrating the antiviral, anti-inflammatory, and barrier-protective effects *in vitro* are strongly indicative of pleiotropic mechanisms of AZM for mitigating viral infection and its consequences. These effects are likely to contribute to the benefits observed in clinical trials of AZM in a number of chronic respiratory diseases.

## 1 Introduction

Respiratory viral infections present a significant global health challenge, often leading to severe complications and exacerbating pre-existing conditions (Xu et al., 2024; Denney and Ho, 2018). The respiratory epithelium serves as the body’s first line of defense against these pathogens, forming a critical physical barrier (Denney and Ho, 2018; Miura, 2019). This protective barrier is maintained by specialized cell-cell junctions, primarily tight junctions (TJs) and adherens junctions (AJs), which are crucial for maintaining barrier integrity, facilitating cell signaling, and providing cellular anchorage (Miura, 2019; Adil et al., 2021). However, respiratory viruses, such as human rhinovirus (HRV), have evolved mechanisms to disrupt these junctional complexes, compromising the epithelial barrier and increasing susceptibility to secondary infections (Sajjan et al., 2008; Looi et al., 2016). Despite the pivotal role of these junctions in host defense, current therapeutic interventions for respiratory viral infections predominantly focus on symptom alleviation and inflammation reduction, with limited strategies aimed directly at enhancing epithelial barrier function (Barnes, 2008).

In recent years, azithromycin (AZM), a macrolide antibiotic, has emerged as a potential therapeutic agent with promising antiviral properties (Khoshnood et al., 2022). While primarily known for its antimicrobial effects, AZM has demonstrated notable anti-inflammatory and immunomodulatory actions (Zarogoulidis et al., 2012). These effects have shown promise in ameliorating symptoms of conditions exacerbated by respiratory viral infections (Menzel et al., 2016; Gielen et al., 2010). Furthermore, foundational work by Gielen et al. demonstrated that AZM can induce antiviral responses, including enhanced interferon production, in bronchial epithelial cells infected with HRV (Gielen et al., 2010). Subsequent studies have suggested that AZM may also help maintain epithelial barrier integrity under conditions of mechanical stress and bacterial infection, indicating a broader capacity for modulating epithelial function (Joelsson et al., 2020; Halldorsson et al., 2010; Ling et al., 2020). The antiviral effects of AZM are hypothesized to arise from its interference with receptor-mediated binding, viral lysosomal escape, and intracellular cell-signaling pathways, as well as from the enhancement of type I and III interferon expression (Parnham et al., 2014). AZM has been shown to decrease mucus production and enhance epithelial barrier thickness of *in vitro* models with respiratory epithelial cells (Slater et al., 2016). It could also diminish the activity of matrix metalloprotease (MMP) following challenge with bacterial lipopolysaccharides, which decreases inflammatory signaling and contributes to the retention of cell integrity and the function of the epithelial barrier (Ribeiro et al., 2009).

Despite the encouraging clinical outcomes associated with AZM (Lakoš et al., 2012; Sun et al., 2023), the specific mechanisms underlying AZM’s beneficial effects during viral infections, particularly its impact on the epithelial barrier itself, remains poorly understood. While research has investigated its role in viral replication, inflammatory modulation, and airway epithelial function, a comprehensive understanding of how AZM affects these processes in pediatric airways requires further elucidation, as they are developmentally and functionally different from adults. This knowledge gap emphasizes the need for additional investigation into the pathways through which AZM exerts its antiviral effects. Given that the epithelium is not only the initial point of pathogen contact but also a crucial component of the early immune response, investigating how AZM affects epithelial function is a critical step towards elucidating its antiviral mechanisms. A closer examination of how AZM modulates the epithelial barrier, particularly in response to viral insults, may yield valuable insights into novel therapeutic strategies targeting epithelial barrier strengthening.

To address this, our study aimed to investigate the effects of AZM pre-treatment on mitigating HRV-1b-induced damage in well-differentiated primary pediatric airway epithelial cells derived from healthy children. Specifically, we focused on AZM’s impact on viral replication, inflammatory responses, the expression and localization of key tight junction proteins (Claudin-1, Occludin, ZO-1), and functional measures of epithelial barrier integrity (transepithelial resistance and paracellular permeability). By examining these aspects in a relevant pediatric cell model, we sought to provide new insights into AZM’s pleiotropic actions and its potential in protecting the airway epithelium during respiratory viral infections.

## 2 Materials and methods

### 2.1 Patients and sample collection

The study was approved by the St John of God Hospital’s Human Research Ethics Committee (Ref #901) and written consent was obtained from each study participant’s legal guardian after being fully informed about the premise and purpose of the study. All experiments were performed in accordance with the relevant committees’ guidelines and regulations. Airway epithelial cells were obtained through trans-laryngeal, non-bronchoscopic brushings of the tracheal mucosa *via* an endotracheal tube of six healthy children after being admitted into hospital for non-respiratory related elective surgery. *Ex vivo* airway epithelial cells were seeded into a pre-coated, irradiated fibroblast seeded tissue culture flask at a density of 1.25 × 10^5^ cells/cm^2^ to establish primary airway epithelial cells (AEC) cultures as previously described (Martinovich et al., 2015). The remainder of the *ex vivo* cells were fractionated out for RNA and protein as previously described (Looi et al., 2018). Children who were diagnosed as having an existing bacterial or viral infection were excluded from the study.

### 2.2 Airway epithelial cell cultures

Primary airway epithelial cells were expanded and maintained as previously described (Martinovich et al., 2015). All submerged monolayer endpoint experiments were performed using bronchial epithelial growth media (BEGM®; LONZA^™^, Switzerland), as described (Looi et al., 2016). For the establishment of air-liquid interface (ALI) cultures, AECs were seeded onto transwell culture scaffolds, pre-coated with collagen type I (ThermoFisher, United States), at a density of 4.5 × 10^5^ cells/cm^2^, with 0.2 mL of 50:50 DMEM/LHC (ThermoFisher, United States), supplemented with: bovine serum albumin (0.5 mg/mL); bovine pituitary extract (10 μg/mL); insulin (0.87 µM); holo-transferrin (0.125 µM); hydrocortisone (0.21 µM); triiodothyronine (0.01 µM); epinephrine (2.7 µM); epidermal growth factor (0.5 ng/mL); trans-retinoic acid (5 × 10^−8^ M); phosphorylethanolamine (0.5 µM); ethanolamine (0.5 µM); zinc sulphate (3 µM); penicillin-G sodium (100 U/mL); streptomycin sulfate (100 μg/mL); Ferrous sulfate (1.5 × 10^−6^ M); Magnesium chloride hexahydrate (×610^−4^ M); Calcium chloride dihydrate (1.0 × 10^−3^ M); Selenium (30 nM); Manganese (1 nM); Silicone (500 nM); Molybdenum (1 nM); Vanadium (5 nM); Nickel (1 nM) and Tin (0.5 nM) (MERCK Life Sciences, Germany). The basolateral chamber received 1 mL of growth medium. Both apical and basolateral media were refreshed daily and when a confluent cell layer was observed, apical medium was removed to initiate the air-liquid interface process. Basolateral media was refreshed every 2 days for a total of 28 days, to allow for maximal cellular polarization and differentiation.

### 2.3 Azithromycin treatment

Confluent submerged monolayer was pre-treated with azithromycin (AZM; Pfizer Inc. United States), prepared in growth media at a final concentration of 0.1, 1 and 10 μM, for initial toxicity assessments over 24 h. Based on these results, 10 µM was selected for all subsequent experiments investigating AZM’s effects on HRV-1b infection. Well-differentiated ALI cultures were similarly pre-treated with AZM in the basolateral chamber to simulate a systemic delivery for 24 h prior HRV-1b infection. Culture supernatant, RNA and cell lysates were collected for downstream experiments conducted to interrogate cytokine mediator production, viral replication and epithelial junctional profiles. Culture scaffolds were also fixed with 50:50 methanol/acetone to assess for epithelial junctional protein expression via confocal laser scanning microscopy.

### 2.4 Rhinovirus and infection

Human rhinovirus minor serotype 1b (HRV-1b; provided by Prof Peter Wark, Monash University, Victoria, Australia) was propagated in HeLa cells and viral titer determined as previously described (Looi et al., 2018). Confluent submerged monolayers and well-differentiated ALI cultures were infected at a 50% tissue culture infectivity dose (TCID_50_) of 1 × 10^5^ TCID_50_/ml. This dose was selected based upon previous work in our laboratory (Looi et al., 2018) to establish a robust infection capable of inducing measurable epithelial responses within a 24-h time frame, with minimal acute cell death which could preclude and thus confound the assessment of AZM’s protective effects. For infection of confluent submerged monolayers and well-differentiated ALI cultures following 24 h AZM pre-treatment, the culture medium containing AZM was removed. For ALI cultures, apical layer was rinsed twice with 0.2 mL of pre-warmed tissue-culture sterile 1×PBS. Viral inoculum (1 × 10^5^ TCID_50_/ml) was then added, (for ALI cultures, viral inoculum was added to the apical chamber) and incubated at 37°C for 2 h to allow for viral adsorption. Following that, viral inoculum was removed, and fresh culture medium containing 10 μM AZM (for ALI cultures, culture media with 10 μM AZM was added to the basolateral chamber) was added, and cultures returned to the incubator for a further 22 h. At 24 h post viral infection, the cell layer was rinsed twice with 0.2 mL of pre-warmed tissue-culture sterile 1×PBS and collected for downstream analysis. Basolateral culture media was also similarly collected.

### 2.5 Quantification of HRV viral copy number

Viral copy number was determined via a two-step RT-PCR as previously described (Sutanto et al., 2011). Briefly, following infection, RNA was extracted from the cells, quantified and reverse transcribed into cDNA using the provided HRV-specific primers. Viral copy was then assessed via Taqman qPCR (Applied Biosystems, United States) using HRV primer-probe mix (PrimerDesign Ltd, United Kingdom) and β actin as a housekeeping gene. The supplied HRV-positive control was serially diluted to generate a standard curve from which viral copy number was subsequently derived.

### 2.6 Quantification of cellular death

Cellular cytotoxicity was analyzed using a CytoTox 96® non-radioactive cytotoxicity assay (Promega, United States) according to the supplied protocol and as previously described (Kicic et al., 2016). The data were represented as measured lactate dehydrogenase (LDH) readings.

### 2.7 Cytokine mediator assay

Cytokine mediator concentrations of interleukin (IL)-1β, −6, −8 and interferon gamma-induced protein (IP)-10 were assessed in collected supernatants using commercial ELISA kits (Bio-Techne, United States) (Kicic et al., 2016). Briefly, each kit was a solid phase sandwich ELISA using monoclonal antibodies specific to the target cytokine. Biotinylated secondary antibodies were used in the detection of the immobilized capture antibodies and streptavidin-peroxidase used as the detection agent. Standard curves were generated using serial dilutions and sample concentrations derived accordingly, with detection limit for each cytokine mediator kit as provided by the supplier.

### 2.8 Quantitative PCR (qPCR)

Gene expression of *intercellular adhesion molecule (ICAM)-1, low density lipoprotein receptor (LDLR), claudin-1*, *occludin* and *zonula occludens-1* (*ZO-1*) and housekeeping gene, *peptidylprolyl isomerase A (PPIA)* was determined via a two-step RT-PCR reaction (Looi et al., 2018) using gene-specific primers (Table 1). Relative gene expression was calculated using the 2^−ΔΔCT^ method by normalization to *PPIA*, due to its uniformity of expression in human epithelial cells.

**TABLE 1:** Oligonucleotide primer sequences.

Gene of interest	Primer	Sequence
*ICAM-1*	Forward	5′-GCAGACAGTGACCATCTACAGCTT-3′
	Reverse	3′-CACCTGGGTCCCTTCTGAGA-5′
*LDLR*	Forward	5′-GACATGAGCGATGAAGTTGG-3′
	Reverse	3′-ATTGCAGACGTGGGAACAG-5′
*Claudin-1*	Forward	5′-GGCAGATCCAGTGCAAAGTC-3′
	Reverse	3′-TCTTCTGCACCTCATCGTCTT-5′
*Occludin*	Forward	5′-AGGCCTGATGAATTCAAACCG-3′
	Reverse	3′-CTGGGTAAAAAGAGTAGGCTGGC-5′
*ZO-1*	Forward	5′-GCCCGTGCCCCGCTCGCTCTC-3′
	Reverse	3′-CCGCCCGCTCCTCACGCCACAG-5′
*PPIA*	Forward	5′-TGAGCACTGGAGAGAAAGGA-3′
	Reverse	3′-CCATTATGGCGTGTAAAGTCA-5′

### 2.9 In cell western™ assay

Confluent cell monolayers which were infected with HRV-1b (1 × 10^5^ TCID_50_/ml) over 24 h were subsequently fixed and assessed for TJ protein expression as previously described (Looi et al., 2016). Briefly, when confluent, culture supernatant was collected and cell monolayer fixed immediately with 3.7% (v/v) formaldehyde for 20 min at room temperature (RT). Cells were then permeabilized (if required) by washing five times with 1×PBS containing 0.1% (v/v) Triton X-100 for 5 min/wash with gentle shaking. This allowed for the determination of total protein expression. Cells were blocked for 90 min at RT using Odyssey Blocking Buffer (LI-COR Biosciences,United States) and subsequently incubated with primary antibodies specific to claudin-1, occludin and ZO-1 (1:200; ThermoFisher, United States) overnight at 4°C. Following this, cells were washed five times at RT in 1×PBS containing 0.1% (v/v) Tween-20 for 5 min/wash with gentle shaking and probed with near-infrared (NIR) fluorescent dye conjugated-secondary antibody (1:800, LI-COR Biosciences, United States) containing cellular nuclear stains DRAQ5 (1:10,000, Biostatus Limited, United Kingdom) and Sapphire700 (1:1000; LI-COR Biosciences, United States) for 1 h at RT with gentle shaking within a dark room. Cells were then washed an additional 5 times in 1×PBS containing 0.1% (v/v) Tween-20. Specific antibody staining for protein expression was then immediately visualized using a two NIR laser imaging system for the excitation of NIR fluorescent dye conjugated-secondary antibodies bound onto the target proteins. The obtained fluorescence was then converted to integrated intensity values through the accompanying image analysis software which was then normalized to cell numbers stained by the cellular nuclear stains (LI-COR Biosciences, United States).

### 2.10 Immunofluorescence confocal microscopy

Well-differentiated ALI cultures were treated with 10 µM of AZM 24 h prior infection with HRV-1b (1 × 10^5^ TCID_50_/ml) for another 24 h. The cells were then fixed in ice-cold 50:50 methanol/acetone for 10 min at −20°C for occludin and ZO-1 staining. Cells were then washed three times at 5 min/wash with 1×TBS followed by non-specific binding in 1×TBS containing 10% (v/v) normal goat serum, 10% (v/v) fetal calf serum and 1% (w/v) bovine serum albumin for 30 min at RT. Cells were then incubated with primary antibodies specific to occludin and ZO-1 (1:200; Invitrogen) for 1 h, followed by six washes at 10 min/wash with 1×TBS. Cells were probed with secondary antibodies (1:1000; Invitrogen) for 1 h in the dark followed by six washes at 10 min/wash with 1×TBS. This was followed by counterstaining with Hoechst 33342 for 5 min. Culture scaffold membranes were mounted with ProLong Gold antifade mountant (ThermoFisher, United States). Control samples were included where the primary antibody was omitted to eliminate any non-specific secondary antibody binding from analysis. Visualization and image capture was performed using a Nikon A1 inverted confocal microscope as previously described (Buckley et al., 2018).

### 2.11 Transepithelial electrical resistance measurement and paracellular permeability

Transepithelial electrical resistance (TER; R_T_) measurement was performed on days 7, 14, 21 and 28 to assess cellular polarization and differentiation as previously described (Looi et al., 2018). Upon achieving optimal differentiation, ALI cultures were infected apically with HRV-1b (1 × 10^5^ TCID_50_/ml) for 24 h and paracellular permeability subsequently assessed. The apparent permeability of the epithelial monolayer to FITC-dextran 4 kDa from the apical to basolateral compartment (P*app*) was then calculated following the general equation: P_*app*_ = (dQ/dt) × (1/AC_0_) where dQ/dt is the steady-state flux, A is the surface area of the membrane and C_0_ is the initial concentration in the donor compartment (Looi et al., 2018).

### 2.12 Statistics

Experiments were performed in duplicates and values are reported as mean ± standard deviation where appropriate. Normality of data was determined using D’Agostino & Pearson normality test. Unpaired t-test was used for parametric data, and Mann-Whitney statistical test for non-parametric data. Correlation or lack thereof was assessed through Spearman non-parametric correlation test. All p values less than 0.05 were considered significant. GraphPad Prism 10.0 software package was used for performing statistical analyses and graphical representation of data.

## 3 Results

### 3.1 Azithromycin pre-treatment does not augment cell viability and cytotoxicity

**FIGURE 1:**
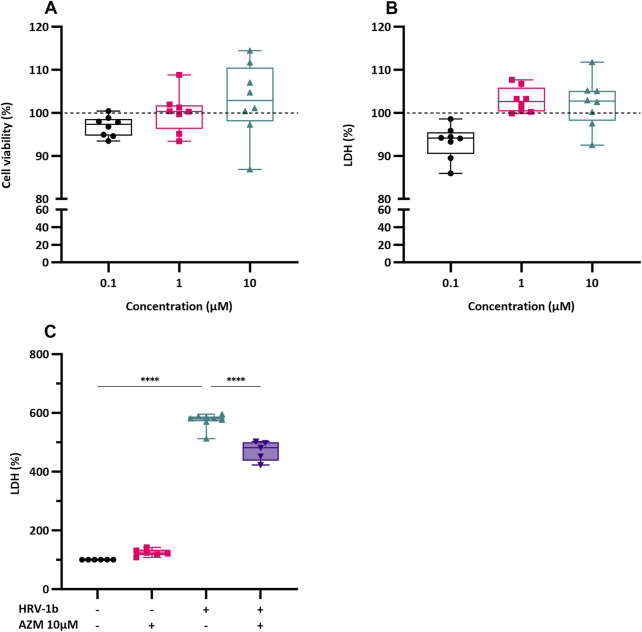
Effect of AZM treatment on cell viability and LDH release in submerged monolayers of crAECs. **(A)** Cell viability was assessed by MTS assay in submerged monolayers of crAECs pre-treated with increasing concentrations of AZM (0.1, 1, and 10 µM) for 24 h. Cell viability is expressed as a percentage to untreated controls (dotted line, ---). **(B)** LDH release was measured as a percentage of total cell lysis in submerged monolayer crAECs under the same treatment conditions to untreated controls (dotted line, ---). Despite modest differences in a dose-dependent response to AZM for both cell viability and LDH, this was not statistically significant. **(C)** LDH levels were measured in the supernatant of submerged monolayer crAECs under different treatment conditions: non HRV-1b-infected, non AZM-treated (−/−), non HRV-1b-infected, AZM-treated (−/+), HRV-1b-infected, non AZM-treated (+/−), and HRV-1b-infected, AZM-treated (+/+). AZM treatment significantly reduced LDH levels in HRV-1b-infected, AZM-treated (+/+) crAECs compared to HRV-1b-infected, non AZM-treated (+/−) controls, indicating reduced cell damage. Note: *n =* 6 with the data represented as mean ± SD, ****p < 0.001, determined using one-way ANOVA with *post hoc* testing for multiple comparisons.

The potential toxic effects of AZM on primary AECs was first determined. Confluent submerged monolayer of primary AECs were treated with AZM (0.1, 1 and 10 µM) for 24 h and subsequently assessed for cell viability and cellular cytotoxicity. Treatment with AZM at the three concentrations did not result in any significant changes in cellular viability compared to untreated controls (Figure 1A; 0.1 µM: 97.03% ± 2.3%; 1 µM: 100.3% ± 4.6%; 10 µM: 102.8% ± 8.7%). Although cell cytotoxicity levels were slightly elevated after treatment with 1 and 10 µM of AZM, these were not statistically different to untreated controls (Figure 1B; 102.9% ± 2.9%, p = 0.2 and 102.2% ± 5.7%, p = 0.6, respectively). As treatment with the three concentrations of AZM did not elicit significant changes in cellular viability or cytotoxicity, subsequent AEC culture experiments were treated with 10 µM of AZM. The effect of AZM on mitigating the cytotoxicity effects of HRV-1b infection was next assessed. Primary AEC cultures were, (i) only treated with 10 µM of AZM (−/+); (ii) only infected with HRV-1b (+/−); or (iii) infected with HRV-1b and also treated with 10 µM of AZM (+/+) (Figure 1C). Treatment with 10 µM of AZM similarly showed no significant increase in cytotoxicity levels, further corroborating initial cytotoxicity assessments (Figure 1C; 124.1% ± 11.6% (−/+); p = 0.07). In contrast, infection with HRV-1b resulted in a significant increase in cytotoxicity levels compared to non-infected, non-treated controls (Figure 1C; 573.8% ± 26.1% (+/−), p < 0.0001). With 10 µM AZM treatment and following HRV-1b infection, cytotoxicity levels were significantly reduced, when compared to HRV-1b infected cells (Figure 1C; 471.6% ± 33.4% (+/+) *versus* 573.8% ± 26.1% (+/−); p < 0.0001).

### 3.2 Azithromycin pre-treatment on viral receptors expression and viral replication

**FIGURE 2:**
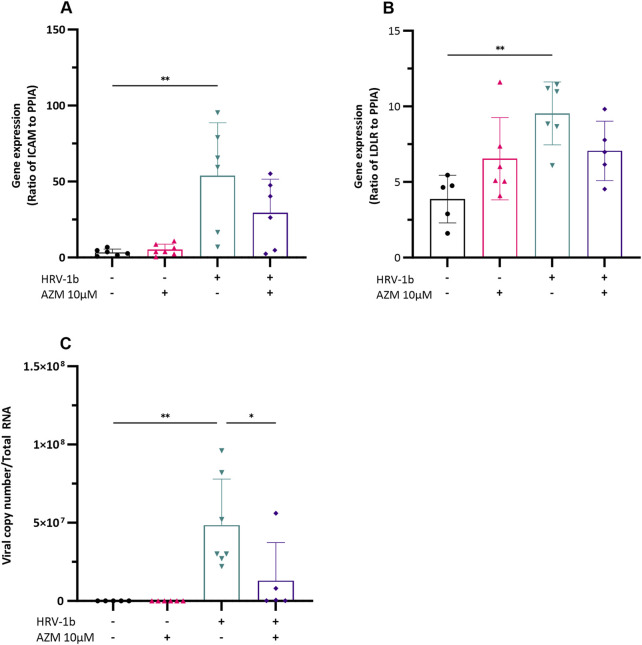
Impact of HRV-1b infection and AZM treatment on receptor expression and viral load in crAECs. **(A)** Ratio of ICAM to PPIA, **(B)** Ratio of LDLR to PPIA, and **(C)** Viral copy number per total RNA were measured in crAECs under different treatment conditions: non HRV-1b-infected, non AZM-treated (−/−), non HRV-1b-infected, AZM-treated (−/+), HRV-1b-infected, non AZM-treated (+/−), and HRV-1b-infected, AZM-treated (+/+). The expression of both ICAM and LDLR relative to PPIA was significantly increased following HRV-1b infection (+/−) compared to paired controls (−/−). AZM treatment (−/+) did not have any effect on either ICAM or LDLR expression and AZM treatment of HRV-1b infected crAECs (+/+) resulted in a modest but statistically mpm-significant decrease in expression of both receptors. Viral copy number, indicative of active viral replication, significantly increased in HRV-1b-infected crAECs (+/−) compared to non HRV-1b-infected, non AZM-treated (−/−) controls. Viral copy number was also significantly reduced in HRV-1b-infected, AZM-treated (+/+) crAECs compared to HRV-1b-infected, non AZM-treated (+/−) controls. Note: *n =* 6 with the data expressed as mean ± SD, */**p < 0.05/0.01, respectively, determined using one-way ANOVA with *post hoc* testing for multiple comparisons.

Confluent submerged monolayer of primary AECs, treated with 10 µM of AZM prior infection with HRV-1b for 24 h were assessed for the viral receptor genes, *ICAM* and *LDLR.* Azithromycin treatment of primary AECs did not significantly augment basal *ICAM* gene expression (Figure 2A; −/+ *versus* −/− respectively). Infection with HRV-1b significantly increased *ICAM* expression compared to non-infected, non-treated controls (Figure 2A; 53.8 ± 34.9 (+/−) *versus* 3.3 ± 2.2 (−/−) respectively; p < 0.01) but despite an overall decrease in *ICAM* expression following AZM treatment in HRV-1b infected cultures, this was not significant (Figure 2A; 29.4 ± 22.1 (+/+) *versus* 53.8 ± 34.9 (+/−); p = 0.1). Similarly, basal *LDLR* expression in AZM treated cultures were not significantly different to non-infected, non-treated controls (Figure 2B; −/+ *versus* −/− respectively). HRV-1b infection significantly increased *LDLR* expression (Figure 2B; 9.5 ± 2.0 (+/−) *versus* 3.8 ± 1.5 (−/−); p < 0.01), however, despite a similar decrease in expression in HRV-1b infected cultures treated with AZM, this was statistically non-significant (Figure 2B; 7 ± 1.9 (+/+) *versus* 9.5 ± 2.0 (+/−); p = 0.2). Viral replication, as indicated by viral copy numbers, was significantly increased following infection and in contrast to *ICAM* and *LDLR* expression levels in infected, treated, cultures, viral copy numbers were significantly lower in the AZM pre-treated, HRV-1b infected cultures, compared to those infected with HRV-1b only (Figure 2C; 1.2 × 10^7^ ± 2.4 × 10^7^ (+/+) *versus* 4.8 × 10^7^ ± 2.9 × 10^7^ (+/−); p < 0.05).

### 3.3 Pre-treatment with azithromycin alters cytokine mediator production

**FIGURE 3:**
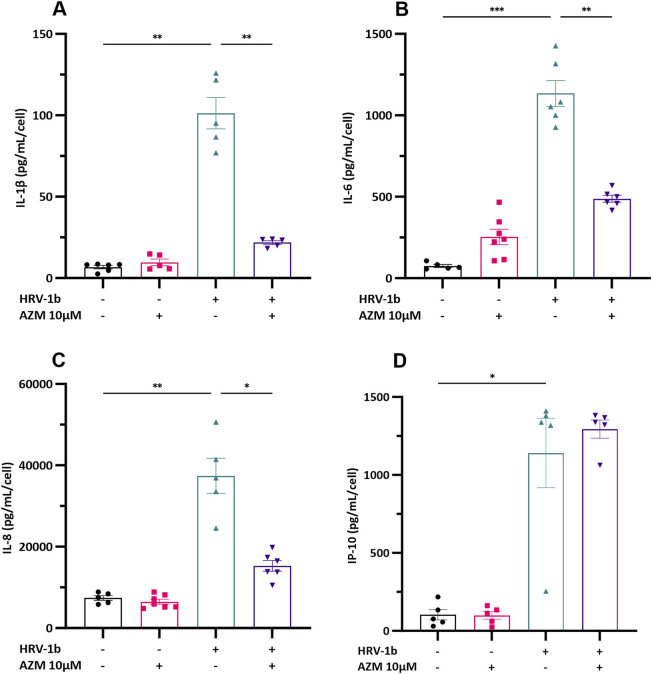
Cytokine production in the supernatant of crAEC of non-diseased children following HRV-1b infection and receiving AZM treatment. Cytokine release was measured in cell culture supernatants using commercial ELISA kits and an in-house time-resolved fluorometry detection system. Inflammatory cytokines: **(A)** IL-1β, **(B)** IL-6, **(C)** IL-8, together with **(D) **IP-10, were not significantly different between non HRV-1b-infected, AZM-treated (−/+) and non HRV-1b-infected, non AZM-treated (−/−) crAECs. **(A–C) ** IL-1β, IL-6 and IL-8 levels, were significantly reduced in the supernatant of HRV-1b-infected, AZM-treated (+/+) crAECs compared to HRV-1b-infected, non AZM-treated controls (+/−). Note: *n =* 6 with the data represented as mean ± SD, */**p < 0.05/0.01 respectively, determined using unpaired *t*-test or Mann–Whitney depending on Gaussian distribution.

To assess whether anti-inflammatory properties of AZM potentially underlie its antiviral effects, primary AECs were treated with AZM 24 h prior infection with HRV-1b and the inflammatory responses subsequently assessed. Production of IL-1β, −6, −8 and IP-10 were then assessed by ELISA (Figures 3A–D). IL-1β, −8 and IP-10 protein levels were not increased following pre-treatment with AZM and despite a modest increase in IL-6 protein levels, this was also not statistically significant, when compared with non-treated, non-infected cells (Figure 3B; −/+ *versus* −/−). Infection with HRV-1b resulted in significant increases in cytokine protein levels compared with non-treated, non-infected cells of: IL-1β (Figure 3A; 101.3 ± 21.6 pg/mL/cell (+/−) *versus* 6.7 ± 2.5 pg/mL/cell (−/−); p < 0.01); IL-6 (Figure 3B; 1134.2 ± 194.4 pg/mL/cell (+/−) *versus* 75.0 ± 20.1 pg/mL/cell (−/−); p < 0.001), IL-8 (Figure 3C; 37,425.4 ± 9632.2 pg/mL/cell (+/−) *versus* 7397.0 ± 1320.7 pg/mL/cell (−/−); p < 0.01) and IP-10 (Figure 3D; 1140.4 ± 496.6 pg/mL/cell (+/−) *versus* 103.4 ± 74.4 pg/mL/cell (−/−); p < 0.05). In primary AECs treated with AZM prior HRV-1b infection compared to HRV-1b infected AECs, significant decrease in cytokine levels was observed in: IL-1β (Figure 3A; 21.8 ± 2.5 pg/mL/cell (+/+) *versus* 101.3 ± 21.6 pg/mL/cell (+/−); p < 0.01), IL-6 (Figure 3B; 488.0 ± 52.9 pg/mL/cell (+/+) *versus* 1134.2 ± 194.4 pg/mL/cell (+/−); p < 0.01), and IL-8 (Figure 3C; 15,310.0 ± 3270.6 pg/mL/cell (+/+) *versus* 37,425.4 ± 9632.2 pg/mL/cell (+/−); p < 0.05). Interestingly, AZM treatment of HRV-1b infected AECs resulted in a modest but not statistically significant increase in IP-10 protein levels compared with HRV-1b infected AECs (Figure 3D; 1294.5 ± 131.4 pg/mL/cell (+/+) *versus* 1140.4 ± 496.6 pg/mL/cell (+/−); p = 0.9).

### 3.4 Effects of pre-treatment with azithromycin on tight junction expression

**FIGURE 4:**
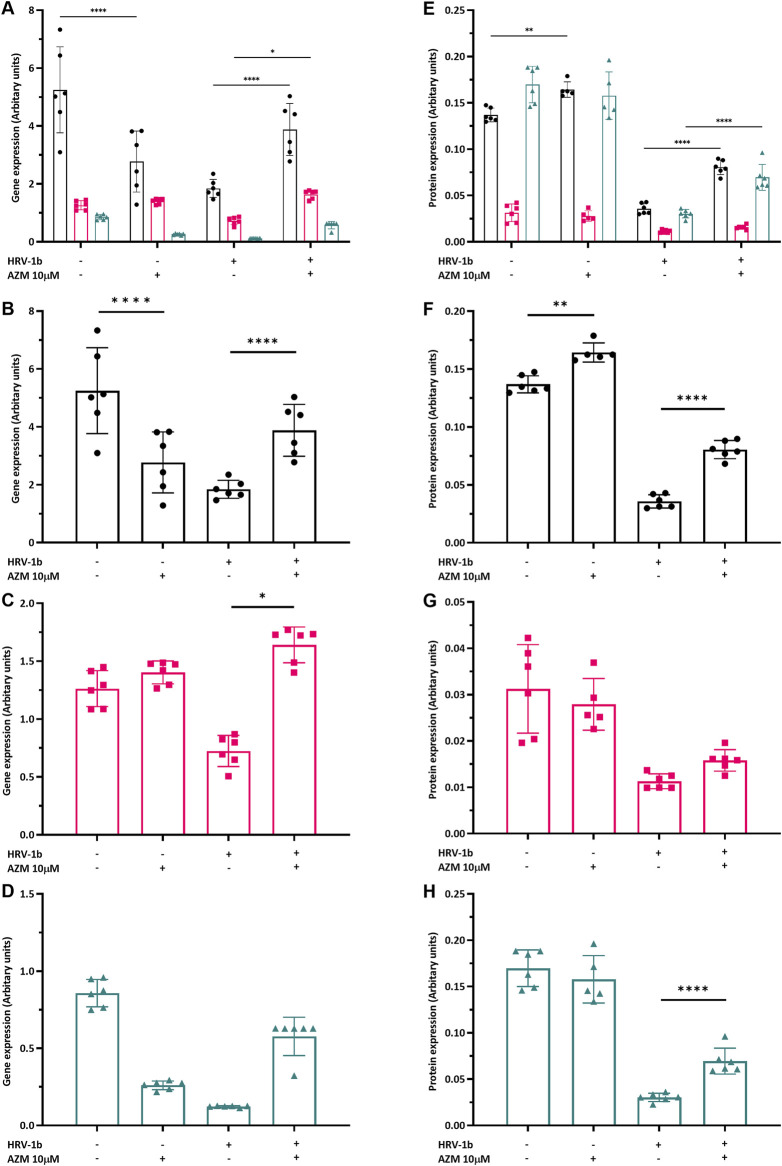
Modulation of tight junction gene and protein expression by HRV-1b infection and AZM treatment in submerged monolayer crAECs. **(A–D)** Gene expression of *claudin-1* (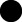), *occludin* (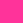), and *Z O -1* (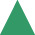) was quantified *via* RT-qPCR in crAECs under different treatment conditions: non HRV-1b-infected, non AZM-treated (−/−), non HRV-1b-infected, AZM-treated (−/+), HRV-1b-infected, non AZM-treated (+/−), and HRV-1b-infected, AZM-treated (+/+). **(E–H)** Protein expression of the same tight junctions under the different treatment conditions was subsequently measured *via* an In-Cell Western^™^ assay. AZM treatment significantly reduced *claudin-1* gene expression but a significant increase in corresponding protein expression was observed. There was no statistically significant modulation of the gene and protein expression of occludin and ZO-1 when compared to non HRV-1b-infected, non AZM-treated controls. AZM treatment of HRV-1b-infected crAECs significantly increased both gene and protein expression of tight junctions, indicating a potential modulatory effect of AZM to restore HRV-1b associated barrier integrity disruption. Note: *n =* 6 with the data expressed as mean ± SD, */**/****p < 0.05/0.01/0.001, respectively, determined using one-way ANOVA with *post hoc* testing for multiple comparisons.

To ascertain if AZM pre-treatment of primary AECs alters tight junction expression and whether this is sustained following infection with HRV-1b, gene and protein expression of claudin-1, occludin and ZO-1 were interrogated. Interestingly, treatment with AZM in non-infected AECs showed a significant 0.5-fold decrease in *claudin-1* gene expression (Figures 4A,B; −/+ *versus* −/−; p < 0.0001) and a 0.4-fold decrease in *Z O -1* gene expression, although this was not significant (Figures 4A,D; −/+ *versus* −/−; p = 0.3). *Occludin* gene expression was not significantly different with AZM pre-treatment, compared to non-treated, non-infected controls (Figures 4A,C; −/+ *versus* −/−; p = 0.9). In HRV-1b infected AECs and treated with AZM, gene expression of both *claudin-1* and *occludin* significantly increased 2.1- and 2.3-fold respectively (Figures 4A–C; +/+ *versus* +/−; p < 0.0001 and p < 0.05 respectively). Despite a 2.9-fold increase in *Z O -1* gene expression, this was not statistically significant (Figures 4A,D; +/+ *versus* +/−; p = 0.9).

Treatment with AZM in non-infected AECs resulted in significant increase in claudin-1 protein expression by 1.2-fold (Figures 4E,F; −/+ *versus* −/−; p < 0.01) but occludin and ZO-1 protein expression were not significantly different to non-treated, non-infected controls. Following HRV-1b infection, claudin-1 and ZO-1 protein expression in primary AECs pre-treated with AZM were 2.2- and 2.3-fold greater compared to non-treated, infected AECs (Figures 4E,F,H; +/+ *versus* +/−; p < 0.0001) and a modest but not statistically significant increase in occludin protein expression (Figure 4G; +/+ *versus* +/−; p = 0.8).

**FIGURE 5:**
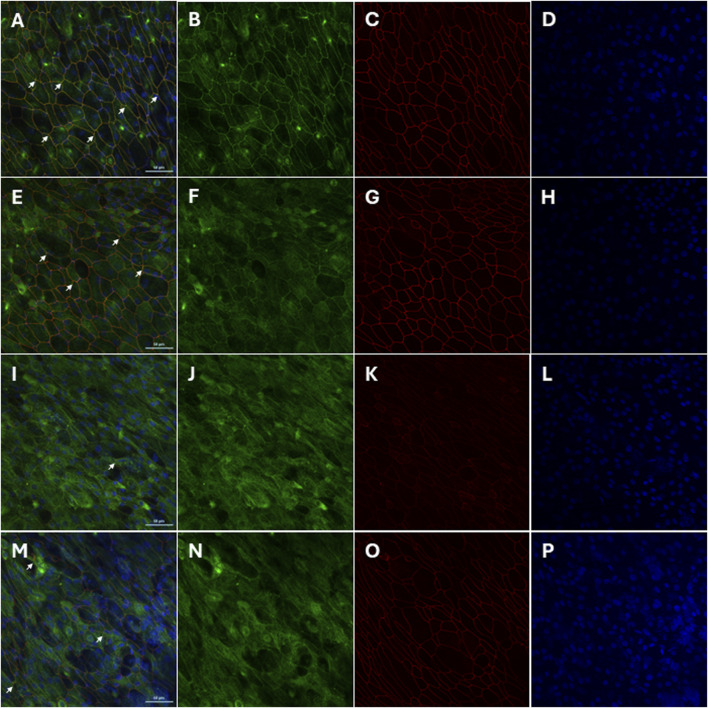
Restoration of tight junction protein localization by AZM treatment in HRV-1b-infected crAECs. The localization of the tight junction proteins ZO-1 (green) and occludin (red) in well-differentiated crAECs was assessed using immunofluorescence staining, with nuclei counterstained with Hoechst 33342 (blue). Panels show representative images of **(A–D)** non-infected, non-treated cells (−/−), **(E–H)** non-infected, AZM-treated cells (−/+), **(I–L)** HRV-1b-infected, non-treated cells (+/−), and **(M–P)** HRV-1b-infected, AZM-treated cells (+/+). In HRV-1b-infected, non-treated cells, a disruption in the junctional expression of occludin and ZO-1 is evident, with a notable decrease in the intensity and organization of these proteins. In contrast, AZM treatment restores tight junction integrity, as shown by the more continuous and organized expression of occludin and ZO-1 at the cell junctions. White arrows indicate junctional expression of the proteins in all panels. Note: Representative images were acquired with a ×40 objective; Scale bar = 50 µm.

Confocal LSM demonstrated that in non-infected, non-treated primary AECs, strong, intense intercellular co-staining, which appears yellow (Figure 5A; white arrows) of both ZO-1 (Figure 5B) and occludin (Figure 5C) were uniformly observed throughout. Nuclei counter-stain showed uniformity of cells (Figure 5D). Pre-treatment with AZM resulted in a markedly less uniform co-staining of both proteins (Figure 5E; white arrows). A less defined and intense staining of intercellular ZO-1 (Figure 5F) and diffused intracellular staining of occludin (Figure 5G) mostly observed. Nucleui counter-stain showed presence and uniformity of cells (Figure 5H). Co-staining intensities for both ZO-1 and occludin becomes markedly less evident following HRV-1b infection (Figure 5I; white arrow). ZO-1 stains are observed to be mostly intracellular and in some intercellular regions (Figure 5J), with intercellular occludin stains observed scattered sparsely across the epithelial layer (Figure 5K). Nuclei counter-stain continues to demonstrate uniformity of cells (Figure 5L). Primary AECs that received AZM following HRV-1b infection showed less diffused co-staining intensities of ZO-1 and occludin (Figure 5M; white arrows). ZO-1 stains continue to be mostly intracellular, with some observed to be intercellular (Figure 5N). Interestingly, occludin staining intensities were markedly stronger and clearly defined within the intercellular regions (Figure 5O). Nuclei counter-stain demonstrated uniformity of AECs (Figure 5P).

### 3.5 Pre-treatment with azithromycin alters epithelial barrier function

**FIGURE 6:**
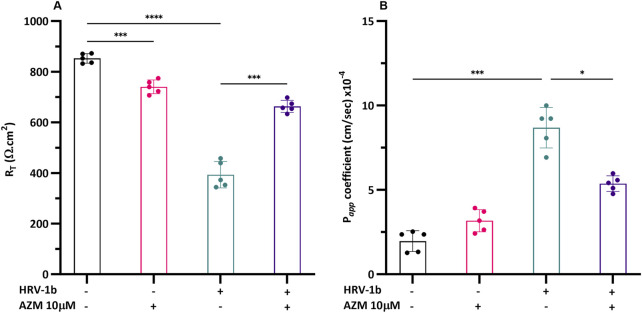
Effect of HRV-1b infection and AZM treatment on barrier function in crAECs. **(A)** Electrical resistance (R_T_) and **(B)** epithelial permeability (P_*app*_) were measured in well-differentiated crAEC under different conditions using an electrical impedance sensing system (R_T_) and FITC-dextran, 4 kDa (P_*app*_). Well-differentiated crAEC were either non-infected, non-treated (−/−), non-infected, AZM-treated (−/+), HRV-1b infected, non-treated (+/−), or HRV-1b infected, AZM-treated (+/+). AZM treatment significantly increased R_T_ in infected, treated (+/+) crAECs compared to infected, non-treated (+/−) controls, suggesting improved barrier integrity. Epithelial permeability was significantly reduced in infected, treated (+/+) crAECs compared to infected, non-treated (+/−) controls, corroborating R_T_ data, indicating improved barrier function. Note: *n* = 5 per condition with data expressed as mean ± SD, */***p < 0.05/0.005, respectively, determined using one-way ANOVA with *post hoc* testing for multiple comparisons.

Further comprehension on the associated implications of altered TJ proteins on epithelial integrity, key to maintaining structural and functional lung defenses, required the interrogation of barrier function in well-differentiated primary AECs. Treatment with AZM in non-infected AECs decreased transepithelial electrical resistance (R_T_) by 0.8-fold, compared to non-treated, non-infected controls (Figure 6A; −/+ *versus* −/−; p < 0.0001) and infection with HRV-1b further reduced R_T_ by 2-fold (Figure 6A; ± *versus* −/−; p < 0.05). R_T_ increased by 1.7-fold in HRV-1b infected AECs treated with AZM (Figure 6A; +/+ *versus* +/−; p < 0.001). To corroborate the resistance measurements, transepithelial permeability across the well-differentiated primary AECs layer was assessed. Treatment with AZM in non-infected AECs did not significantly increase epithelial permeability when compared to non-treated, non-infected controls. In contrast, epithelial permeability in primary AECs infected with HRV-1b increased by 4.4-fold, when compared to non-treated, non-infected controls (Figure 6B; −/+ and ± respectively *versus* +/−; p < 0.001). HRV-1b infected AECs treated with AZM resulted in a significant 0.6-fold decrease in permeability when compared to primary AECs infected with HRV-1b only (Figure 6B; +/+ *versus* +/−; p < 0.05).

## 4 Discussions

The present study aimed to elucidate the impact of azithromycin (AZM) on the modulation and reorganization of tight junction (TJ) proteins following epithelial damage induced by human rhinovirus (HRV)-1b infection. Our findings indicate that pre-treatment with 10 µM AZM, a concentration achievable in respiratory secretions and used in prior *in vitro* assessment (Baumann et al., 2004), mitigated HRV-1b induced epithelial disruption through multiple actions. While our initial assays showed a statistically significant albeit minor increase in baseline LDH release at 1 and 10 µM AZM compared to controls, this was minimal compared to virus-induced cytotoxicity and did not significantly affect overall cell viability. Importantly, 10 µM AZM significantly reduced virus-induced cytotoxicity post-HRV-1b infection. Furthermore, consistent with earlier findings demonstrating AZM’s antiviral properties against HRV (Gielen et al., 2010; Schögler et al., 2015), we observed that AZM treatment significantly reduced HRV-1b replication in pediatric airway epithelial cells. Interestingly, this occurred without significantly altering the expression of viral receptors ICAM-1 and LDLR, suggesting AZM may interfere with viral replication steps downstream of initial binding and entry, or enhance intracellular antiviral defenses. Similarly, we also demonstrated AZM’s ability to modulate inflammatory responses and protect epithelial barrier integrity. AZM treatment led to significant reductions in the production of pro-inflammatory cytokines IL-1β, IL-6, and IL-8, which are often elevated during viral infections and contribute to epithelial damage. These observations contrast with previous reports (Gielen et al., 2010; Schögler et al., 2015) showing no significant effect of AZM treatment on HRV-1b induced cytokine protein levels. These differences may be attributed to variations in the experimental model (pediatric-derived non-diseased primary tracheal epithelial cells *versus* commercially derived or diseased bronchial epithelial cell lines), AZM concentrations (10 µM *versus* 50 µM), or the duration of viral adsorption (2 h *versus* 1 h). Importantly, the observed reduction in cytokine-mediated inflammation suggests that AZM plays a multifaceted role not only in antiviral defense but also in mitigating inflammatory damage, which is crucial for preventing exacerbations in chronic respiratory conditions. Interestingly, we observed a non-significant increase in IP-10 expression following HRV-1b infection and AZM treatment, a finding consistent with Schögler et al. (Schögler et al., 2015), which suggests that the role of IP-10 in AZM treatment remains complex, with some studies suggesting an enhancement of immune responses (Schögler et al., 2015; Taima et al., 2006), while others report a suppressive effect (Kuo et al., 2019). This indicates that AZM could have alternative, pathway-specific immunomodulatory actions, rather than just broad suppression effects alone. Further research is needed to clarify the role of AZM in modulating IP-10 and its associated upstream signaling components like STAT1 activation and its implications for host antiviral defense mechanisms during HRV infections.

A key contribution of this study is the demonstration of AZM in enhancing barrier integrity by increasing the expression of key TJ proteins, specifically claudin-1 and occludin, following HRV-1b infection. This effect was accompanied by improved transepithelial resistance and reduced epithelial permeability, indicating enhanced epithelial cohesion and reduced susceptibility to further insults. These findings are consistent with studies suggesting that AZM can bolster the physical barrier properties of epithelial cells under stress conditions, thereby highlighting its role as a protective agent beyond its conventional antimicrobial action (Halldorsson et al., 2010; Asgrimsson et al., 2006; Arason et al., 2019). Strengthening of the epithelial barrier is paramount in respiratory diseases, as a compromised epithelium can facilitate pathogen and/or allergen entry, leading to increased susceptibility to subsequent infections and chronic exacerbations. Another significant finding is the differential effect of AZM on TJ protein expression in infected *versus* non-infected cells. While AZM decreased *claudin-1* gene expression in non-infected cells, it increased *claudin-1* and *occludin* expression following HRV-1b infection. This dynamic modulation suggests that AZM’s effect on TJ proteins may depend on the cellular context, potentially acting agonistically when the epithelial barrier is compromised. To our knowledge, this study is the first to report TJ modulation following HRV-1b infection and AZM treatment, representing a novel discovery that warrants further investigation into the underlying mechanisms. AZM’s ability to restore TJ integrity may be particularly beneficial in conditions such as asthma, where disrupted TJs are associated with increased viral infiltration and exacerbated disease severity. Such a mechanism could be particularly beneficial in disease states, where maintaining or restoring epithelial integrity is critical for reducing disease severity and preventing secondary infections.

While this study provides valuable insights, certain limitations must be acknowledged. We used a well-differentiated *in vitro* model of lower airway epithelial cells, which, despite its advantages, lacks the complexity of *in vivo* models that include immune cells such as neutrophils and macrophages. These immune cells play pivotal roles in antiviral responses, and it is possible that AZM exerts effects independent of airway epithelial cells. Previous studies have shown that AZM reduces pro-inflammatory cytokine expression by alveolar macrophages (Yang, 2020) and suppresses neutrophil activation and chemotaxis (Feola et al., 2010). Furthermore, the precise molecular mechanisms by which AZM influences TJ pathways remain unclear, and further research is essential to elucidate these pathways and develop novel therapeutic strategies targeting barrier restoration. Additionally, this study focused solely on epithelial cells derived from healthy children, leaving a gap in our understanding of how AZM may affect epithelial responses in individuals with respiratory diseases, such as asthma or chronic obstructive pulmonary disease (COPD), where epithelial barrier dysfunction is more pronounced. Caution should be exercised when extrapolating these findings to diseased populations, and future studies should evaluate AZM’s efficacy and safety in diseased epithelium.

In conclusion, our study provides compelling evidence that AZM can mitigate HRV-1b-induced epithelial damage through combined antiviral, anti-inflammatory actions, as well as barrier-enhancing actions. These pleiotropic effects position AZM as a promising candidate for therapeutic strategies aimed at fortifying the epithelial barrier during respiratory viral infections. Further research is needed to explore the underlying mechanisms in more detail and to validate these findings *in vivo*, potentially paving the way for novel interventions in respiratory viral infections in vulnerable pediatric populations.

## Data Availability

The original contributions presented in the study are included in the article, further inquiries can be directed to the corresponding author.
